# Toward the consensus of definitions for the phenomena of antifungal tolerance and persistence in filamentous fungi

**DOI:** 10.1128/mbio.03475-24

**Published:** 2025-02-25

**Authors:** Jorge Amich, Michael Bromley, Gustavo H. Goldman, Clara Valero

**Affiliations:** 1 Mycology Reference Laboratory, National Centre for Microbiology, Instituto de Salud Carlos III (ISCIII), Madrid, Spain; 2 CiberInfec ISCIII, CIBER en Enfermedades Infecciosas, Instituto de Salud Carlos III, Madrid, Spain; 3 Manchester Fungal Infection Group (MFIG), Division of Evolution, Infection, and Genomics, Faculty of Biology, Medicine and Health, University of Manchester, Manchester, United Kingdom; 4 Faculdade de Ciências Farmacêuticas de Ribeirão Preto, Universidade de São Paulo, Ribeirão Preto, Brazil; 5 National Institute of Science and Technology in Human Pathogenic Fungi, São Paulo, Brazil; Hebrew University of Jerusalem, Rehovot, Israel

**Keywords:** antifungal tolerance, antifungal persistence, antifungal drugs, filamentous fungi

## Abstract

Antifungal drug tolerance and persistence are being increasingly recognized in fungal pathogens. Accordingly, more and more research is being carried out to characterize and understand these phenomena. However, the terminology and methodology employed in the fungal community lack consensus, particularly for filamentous fungi, as they present further complexities when compared to single-celled microorganisms. Hence, with the aim to ensure consistency in the literature, in this Perspective article, we propose tailored definitions for tolerance and persistence in filamentous fungi and suggest methods to detect and investigate these phenomena in the laboratory.

## PERSPECTIVE

Antimicrobial tolerance and persistence are definitions that describe the ability of microorganisms to survive the action of antimicrobial drugs for extended periods. These phenomena are distinct from antimicrobial resistance and heteroresistance, which lead to an increase in the minimum inhibitory concentration (MIC) of the drug (Table 1). Nevertheless, they require that pathogenic microbes survive at supra-MIC concentrations for extended periods, which may have implications for clinical treatment of infections.

**TABLE 1:** Adjusted definitions for filamentous fungi^*a*^

Phenomenon	Bacterial definition	Tailored for filamentous fungi
Resistance	Stable capacity of an isolate to grow normally in the presence of high concentrations of the drug, above the MIC value defined for the antimicrobial based on the clinical breakpoint or epidemiological cutoff value. This capacity is based on specific genetic sequence(s) (intrinsic or acquired) in the genome (1–3).	The same definition for filamentous fungi
Heteroresistance (monoclonal unstable)	Transient capacity of a subpopulation of cells of a susceptible isolate to increase the MIC in the presence of the drug, without a modification in the genome sequence (4)	The same definition for filamentous fungi
Tolerance	Ability of all cells of a susceptible, genetically isogenic strain to survive for extended periods in the presence of cidal drug concentrations that are greater than the MIC (1)	Capacity of all or high proportion of spores of a susceptible isolate to survive for extended periods in the presence of cidal drug concentrations that are greater than the MIC
Persistence	Ability of a small subpopulation of cells of a susceptible, genetically isogenic strain to survive for extended periods in the presence of cidal drug concentrations that are greater than the MIC (1, 5)	Capacity of a small fraction of spores of a susceptible isolate to survive for extended periods in the presence of cidal drug concentrations that are greater than the MIC

^*a*^ MIC, minimum inhibitory concentration.

In bacteria, these phenomena have been intensively studied for more than two decades. As a result, numerous underlying mechanisms have been described (6), and the gathered evidence has led to a consensus that tolerance and persistence can be a predisposing factor for the development of resistance, leading to treatment failure (7, 8). Studies of tolerance and persistence in pathogenic fungi and their relevance for the outcome of infection are still scarce.

In bacteria, there have been community efforts to unify the definitions for antibiotic tolerance and persistence and to establish standardized protocols to describe these phenomena (1, 5). Antibiotic tolerance describes an isogenic cell population that is able to survive for extended periods in the presence of a concentration of a bactericidal drug that exceeds the MIC. As a result, the time required to kill a large fraction of the cells (typically defined with a threshold of 99%), or minimum duration for killing (MDK_99_), in a drug-tolerant strain is longer than that in a non-tolerant isolate. In persistence, only a small subpopulation (0.01%) of “persister” cells are able to survive for extended periods in the presence of a supra-MIC of a bactericidal drug. Therefore, persister isolates are characterized by biphasic killing curves in the presence of a given drug, where initial killing is similar to a susceptible isolate (MDK_99_ may be equal), but higher levels of killing (99.99%) take substantially longer (1, 5).

The terms tolerance and persistence are increasingly being used to define behaviors of pathogenic fungi in response to antifungal drugs; however, the phenomena under investigation do not meet the definitions of these criteria in bacteria. Specifically, in bacteria, the terms tolerance and persistence are not applied to drugs that are bacteriostatic, as by definition these drugs halt growth but do not rapidly kill bacteria (i.e., the MDK_99_ is already prolonged or cannot be determined) (9). In yeasts, however, the term tolerance is used to describe the ability of cells to grow at slow rates at supra-MIC concentrations of both fungistatic (10–14) and fungicidal drugs (10, 15).

We therefore believe that there is a need to define guidelines and unify definitions to ensure consistency and avoid confusion when studying tolerance and persistence in fungi. Defining terminology in filamentous fungi presents further complexities when compared to single-celled bacteria and yeasts as they have obligate life cycles that include distinct morphotypes that exhibit unique characteristics. Hence, in this Perspective article, we aim to provide useful guidelines to work with antifungal tolerance and persistence and provide definitions for these phenomena in filamentous fungal pathogens.

Arguably, it would be physiologically more relevant to study the effect of antifungals on hyphae, as this is the morphotype that is found inside the infected hosts. However, working with hyphal networks (mycelia) in the laboratory presents important obstacles that limit our ability to standardize assays. Most notably, it is impractical to accurately quantify cell numbers in mycelia; hence, assays that rely on quantitative outputs, such as MDK_99_, cannot be calculated. Similarly, quantifying and normalizing homogeneous cultures of young hyphae that have yet to form a network or early germinated spores (germlings) are challenging as germination of these morphotypes from spores is asynchronous (16). Accordingly, the common practice in antifungal research is working with asexual spores (conidia). In fact, the Clinical and Laboratory Standards Institute and European Committee for Antimicrobial Susceptibility Testing standardized protocols, used worldwide to detect antifungal resistance in filamentous fungi, are performed with conidia (17, 18), even if it has been reported that hyphae may have higher MICs than conidia (19, 20) (an observation that we have confirmed in our own laboratories). Similarly, we propose to employ conidia as the standard method to detect tolerance and persistence in filamentous fungi. In this manner, the methods would be harmonized with those to detect antifungal resistance, and thus, consistency would be maintained.

Here we suggest that the terms persistence and tolerance against filamentous fungal conidia should be described in relation to the killing effects of antifungals. However, this definition necessitates further clarification of the definition of antifungal killing. Currently, the stringent gold-standard criterion for defining a fungicidal drug has been exported directly from terminology defined for bacteria (21). To be defined as a bactericidal drug, 99.9% (or 3-log-unit decrease) of cells need to be killed within 24 h of exposure. However, as the vast majority of antimicrobial agents (including antibiotics) require “active growth” to achieve their effect, it will always be challenging to achieve this threshold for fungal spores, a morphotype which is considered to be in a dormant state. Therefore, *in vitro* determination of cidality is often not achieved for compounds like azoles (that require growth for their activity [22, 23]) in pathogens such as *Aspergillus fumigatus* that exhibit asynchronous conidial germination (16). Nevertheless, it is clear that voriconazole can kill both *A. fumigatus* hyphae and conidia (20, 23–26), with observed efficiencies of >95% killing for conidia within 24 h (20, 24, 26–28), which has been proven sufficient to detect persistence (27). This range of killing seems to be achieved for various fungicidal drugs on different species of filamentous fungi (29–31). Therefore, we suggest that the action of a specific drug on the spores of a particular species of a filamentous fungus should be analyzed before investigating the phenomenon of tolerance and persistence. We propose that a drug that can kill ≥90% of spores in 24 h (MDK_90_ ≤ 24 h) of all isolates of a given species should be considered fungicidal.

**Fig 1:**
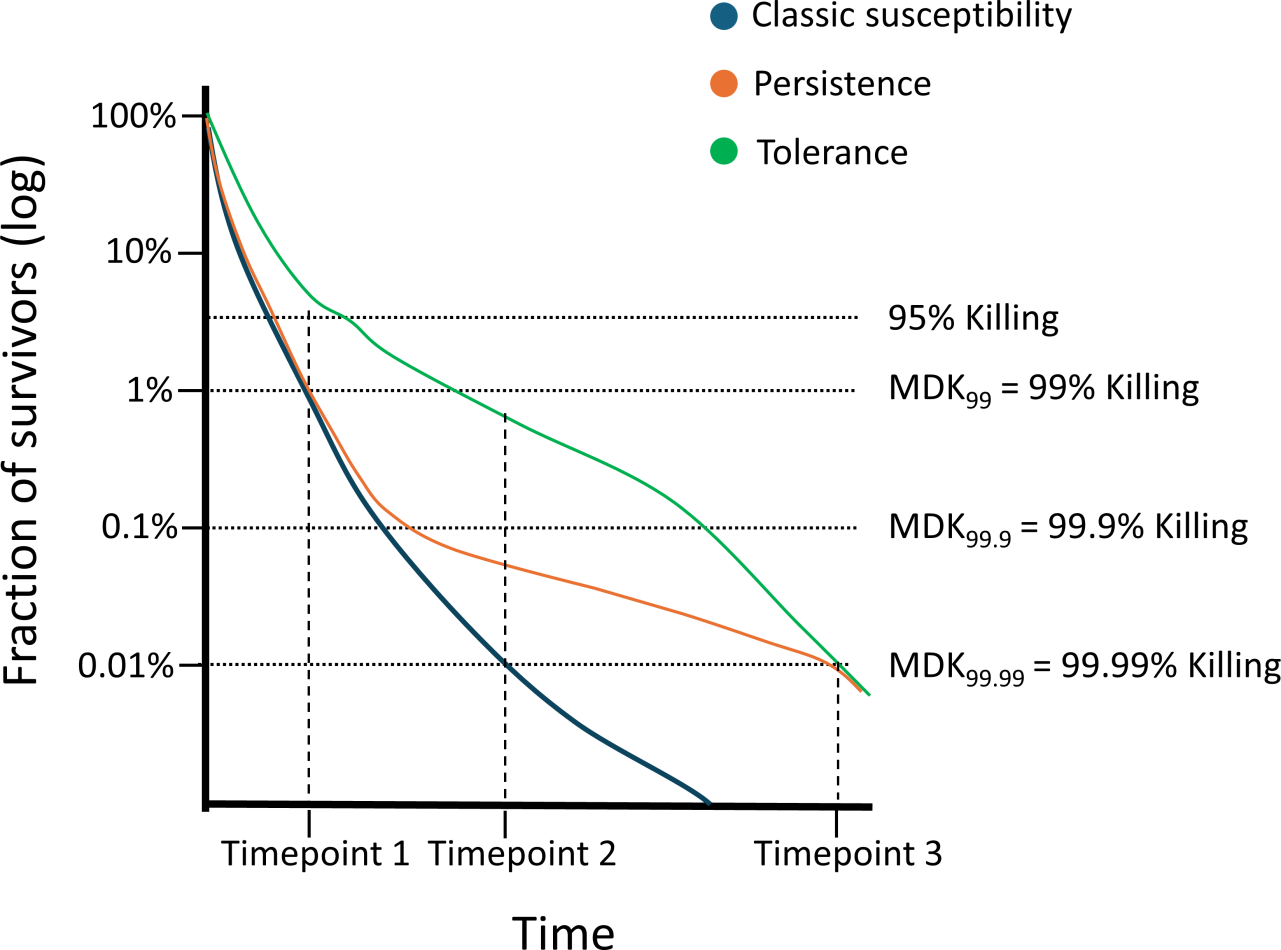
Exemplary curve of the killing dynamics of spores to differentiate tolerance and persistence from general susceptibility. To evaluate the cidality of the drug, >90% killing should be reached within 24 h (MDK_99_ ≤ 24 h). For tolerant strains, MDK_99_, MDK_99.9_ and MDK_99.99_ would be longer than for a susceptible isolate. For persister strains, MDK_99_ would be indistinguishable from a non-persister isolate, but MDK_99.9_ and MDK_99.99_ are longer than for a non-persister isolate.

Antifungal tolerance would be defined as the capacity of all or a high proportion of spores of a susceptible isolate to survive for extended periods in the presence of drug concentrations that are greater than the MIC. Effectively, this would mean that the MDK_99_, MDK_99.9_, and MDK_99.99_ of a tolerant isolate are longer than those for a non-tolerant isolate (Fig. 1).

Persistence would be defined as the capacity of a small fraction of spores (typically <0.1%) of a susceptible isolate to survive for extended periods in the presence of drug concentrations that are greater than the MIC. Effectively, this would mean that the MDK_99_ of a persister isolate is indistinguishable from a non-persister isolate, but the MDK_99.9_ and MDK_99.99_ are longer than those for a non-persister isolate (Fig. 1).

**Fig 2:**
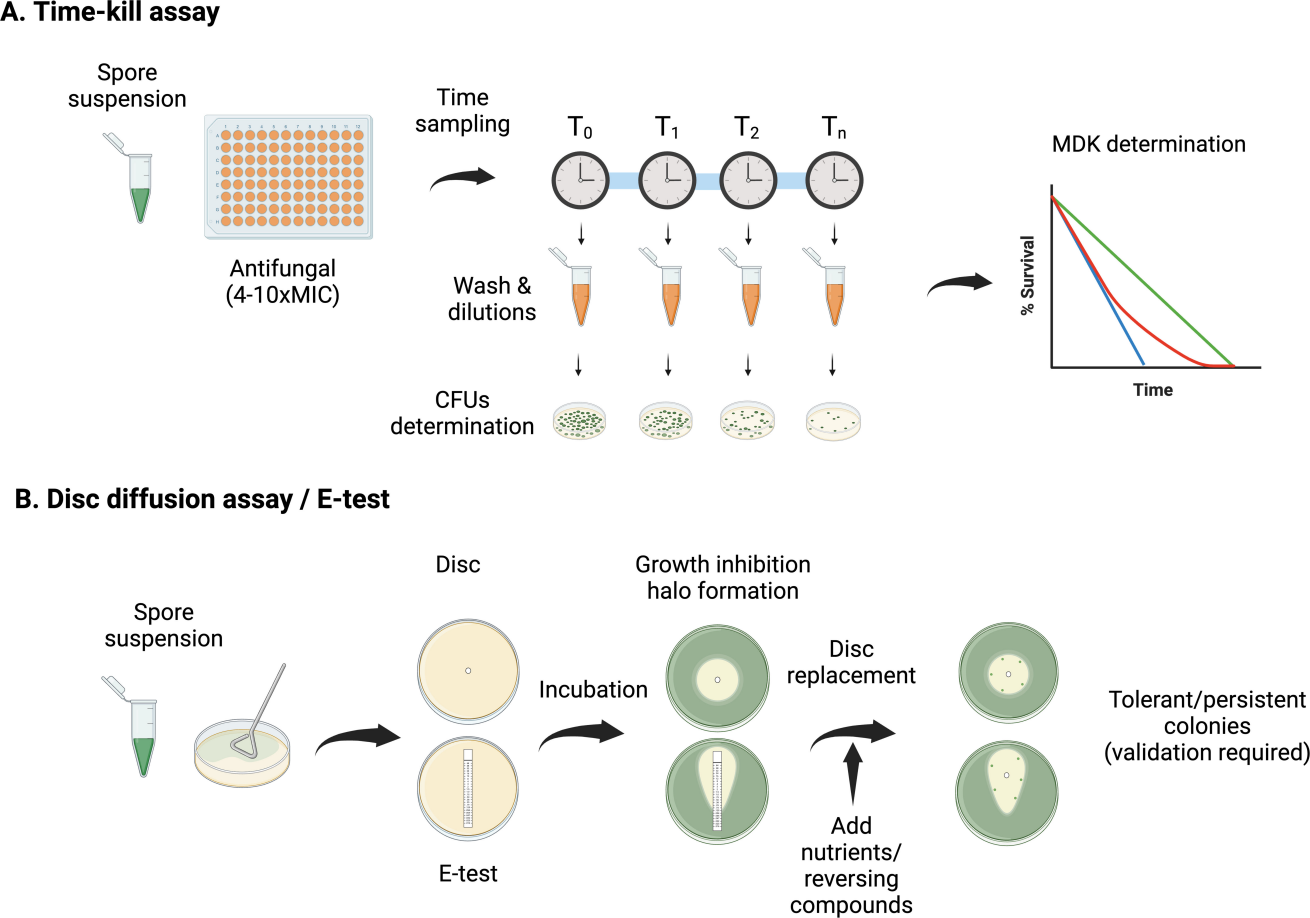
Methods for antifungal tolerance and persistence detection in filamentous fungi. (A) Time-kill assay. Conidia from the filamentous fungi of interest are exposed to supra-MIC concentrations (4–10 times the MICs are recommended) of a given antifungal in the culture media of choice. Samples are taken at different time points, washed, and diluted (vortexing and visual confirmation of the absence of clumps under the microscope are recommended), followed by plating on drug-free solid media. CFUs can be then enumerated after an additional incubation period and MDK values assessed to differentiate tolerance or persistence from general susceptibility. (B) Disk diffusion assay or E-test. Conidia from the filamentous fungi of interest are plated in drug-free solid media of choice, and a disk impregnated with a given antifungal or an E-test stripe is placed at the center of the plate. After incubation, a growth inhibition halo around the disk will be observed. At this point some colonies can be observed within the inhibition halo; however, we recommend replacing the disk by a new one with a compound that reverts the cidal effect of the antifungal tested (if available) or with fresh medium. After an additional incubation, the delayed killing observed in strains exhibiting tolerance or persistence will be seen as colonies growing within the inhibition halo proximal to the disk. The killing dynamics of those positive colonies detected should then be characterized using a time-kill assay to differentiate tolerance from persistence and further confirming the obtained results.

To detect and quantify tolerance and persistence, methods derived from the bacterial field should be used. We propose that the gold-standard method should be the use of killing curves in liquid media in the presence of very high concentrations of the fungicidal antifungal (recommended 4–10 times the MIC). To calculate the percentage of killing, the spores were treated with antifungal over a time course and then washed and plated on drug-free rich medium to count colony forming units (CFUs) (Fig. 2). Time to reach MDK_99_, MDK_99.9_, and MDK_99.99_ can then be used to define if a strain is tolerant or persister (Fig. 1). It is important to remark that the exact time points to measure CFUs and the thresholds should be determined for each drug–fungal species interaction.

An additional method to detect tolerance and persistence could be the use of diffusion assays, disk diffusion, or E-test; although these methods are only semi-quantitative, they can be useful for screening large collections of strains. In some cases, active growth in the halo of inhibition can already be detected after the period of incubation with the drug (27), although we have observed that not all persister strains display this active growth. To properly detect tolerance or persistence, the disk or E-test must be substituted, after the period of incubation with the drug, for either a disk containing a compound that reverses the action of the drug (if available) or fresh medium (to provide new nutrients). This will allow the spores that have survived the action of the drug to germinate and grow to be detected (27, 32) (Fig. 2). Nevertheless, in this case, it can be challenging to distinguish between tolerance and persistence, as the quantification on solid media is imprecise (the number/percentage of spores that are located inside the inhibition halo cannot be easily determined). Consequently, positive results should always be validated through the use of killing curves and MDK determination as the gold-standard method for tolerance and persistence determination.

Antifungal drugs that do not reach the 90% killing threshold in 24 h should be considered fungistatic, and therefore, tolerance and persistence definitions are not applicable to these. The fungistatic nature of drugs is due to the inherent ability of a filamentous fungal species to withstand its action. A perfect example is the echinocandins, which do not kill *A. fumigatus* conidia. In fact, filamentous fungi usually can grow substantially in the presence of fungistatic drugs, a phenomenon that has been classically termed trailing or paradoxical effect growth (33).

Hence, we propose tailored definitions for the phenomena of tolerance and persistence in filamentous fungi (Table 1), which we hope can serve to unify the terminology, providing consistency for future studies and facilitating the understanding within this field. Additionally, we aim for our considerations to serve as valuable guidance for the best approaches for detecting and investigating tolerance and persistence in future research.
